# The Effect of Long-Term Passage on Porcine SMCs’ Function and the Improvement of TGF-β1 on Porcine SMCs’ Secretory Function in Late Passage

**DOI:** 10.3390/foods12142682

**Published:** 2023-07-12

**Authors:** Yan-Yan Zheng, Ze-Nan Hu, Zheng Liu, Yi-Chen Jiang, Ren-Peng Guo, Shi-Jie Ding, Guang-Hong Zhou

**Affiliations:** 1 National Center of Meat Quality and Safety Nanjing, Key Laboratory of Meat Processing and Quality Control, Key Laboratory of Meat Processing, Nanjing 210095, China; 2020208037@stu.njau.edu.cn (Y.-Y.Z.); 2021121009@stu.njau.edu.cn (Z.-N.H.); 2021208036@stu.edu.njau.cn (Z.L.); 2020208036@stu.njau.edu.cn (Y.-C.J.); rpguo@njau.edu.cn (R.-P.G.); shijieding@njau.edu.cn (S.-J.D.); 2 College of Food Science and Technology, Nanjing Agricultural University, Nanjing 210095, China

**Keywords:** cultured meat, large-scale cultivation, SMCs, TGF-β1, extracellular matrix proteins

## Abstract

Cultured meat is one of the meat substitutes produced through tissue engineering and other technologies. Large-scale cell culture is the key for cultured meat products to enter the market. Therefore, this study is aimed to explore the effect of long-term passage in vitro on smooth muscle cells (SMCs) and the effect of transforming growth factor-β1 (TGF-β1) on SMCs in the late passage. Multiple passages lead to the decline of the proliferation rate of SMCs in the proliferation stage and the differentiation ability in the differentiation stage. Transcriptome results showed that the ECM pathway and aging-related signaling pathways were significantly up-regulated in the late passage period. TGF-β1 did not promote SMCs of late passage proliferation at the proliferation stage but promoted the gene and protein expression of collagen as the main protein of the extracellular matrix proteins at the differentiation stage. In addition, proteomic analysis revealed that TGF-β1 promoted the expression of cell adhesion molecules which activate the Hippo signaling pathway and the HIF-1 signaling pathway and further promoted the production of collagen-containing extracellular matrix proteins. This could provide ideas for large-scale production of cultured meat products using SMCs.

## 1. Introduction

The rapidly growing world population will increase meat consumption in the future. With the improvement of the consumption level of meat products, the expansion of traditional animal husbandry and the breeding industry has been promoted [1,2]. Intensive animal husbandry has brought a series of problems, such as land use, energy consumption, greenhouse gas emissions and animal welfare [2,3,4,5,6]. Therefore, traditional livestock breeding for meat production may be unsustainable in the future, and technological innovation is needed to meet the growing global demand for meat consumption while protecting the environment. In order to solve a series of problems caused by meat production, people are also searching for meat substitutes to replace traditional meat, such as plant protein, insect protein, fungal protein, etc. [7]. However, due to people’s inherent desire for meat-flavored and -textured foods, researchers have further studied techniques that may produce animal proteins.

As one of the emerging disruptive technologies for traditional animal husbandry, cultured meat has attracted wide attention due to its advantages of traceability, food safety, greenness and sustainability [8]. Cultured meat technology is a technology of cultivating cells in vitro and inducing cell differentiation to produce animal proteins for meat production. Although the technology of cultured meat has developed rapidly in recent years, there are still many technical difficulties, including the functional maintenance of cultured cells in vitro [9], the development of a serum-free culture medium [10], a three-dimensional cell culture to construct simulated meat [11], etc. In addition, the reagents currently used to produce cultured meat products have food safety risks, and the subsequent realization of organic production to reduce food safety risks is the key to achieving cultured meat on the table to pass the examination of government. Hence, there is also a need to develop more seed cells that can be used to cultured meat production. Many types of cells are incorporated into the preparation of cultured meat, including muscle stem cells [12], adipocytes [13], fibroblasts [14], etc. These cells will produce muscle protein, fat, extracellular matrix protein and other components after differentiation, which endow the cultured meat products with good quality attributes. In combination with tissue engineering technology, the texture of products prepared by inducing differentiation of these cells in vitro is quite different from that of traditional meat products [15]. As one of the three major muscle types, smooth muscle has dense connective tissue network and high content of collagen, so the shear force of meat by-products composed of smooth muscle is far greater than that of skeletal muscle [16]. SMCs, which make up smooth muscle tissue, produce both muscle proteins [15] and extracellular matrix proteins [17], which are considered a good choice for seed cells in cultured meat.

SMCs have two phenotypes: contractile and synthetic. In vivo, highly differentiated SMCs are contractile phenotypes that are at rest and heavily express contractile proteins [18]. In vitro, SMCs gradually changed from contractile cells to synthetic cells and then began to migrate and proliferate and secrete some extracellular matrix proteins [19,20]. If SMCs properties are used as seed cells for cultured meat production, the secretory and proliferative properties of SMCs in long-term culture in vitro need to be further evaluated. In addition, the large-scale cultivation is essential if cultured meat is to reach the market in the next few years [21]. From the initial two-dimensional culture to the later three-dimensional culture such as bioreactors, many meat culture teams have been optimizing biological processes through innovative technologies [22]. Since the cells themselves cannot proliferate indefinitely in vitro, the amount of cells harvested during the expansion culture process will be affected by the characteristics of the cells themselves [23]. Large-scale cell expansion means that SMCs need to be subcultured in vitro many times, and cells are bound to be aged. Transforming growth factor β1 (TGF-β1) is the principal pro-fibrotic factor [24,25], which is considered to be the main acting factor in fibrotic diseases. In addition to the classical TGF-β/Smad signaling pathway, upregulation of interleukin-11 (IL-11) is a major transcriptional response to TGF-β1 treatment and is required for its profibrotic effects [26,27]. IL-11 and its receptor (IL11RA) are specifically expressed in fibroblasts, where they drive an atypical ERK-dependent autocrine signal to promote fibroblast synthesis [28,29]. Current studies on the promoting effect of TGF-β1 on fibrin production by cells have focused more on pathological studies.

Therefore, this research aims to investigate the effects of long-term passage on SMCs and then explore the effects of TGF-β1 on the function of SMCs in the later passage and further explore its action pathways. This should help improve the properties of SMCs as seed cells for cultured meat production and provide theoretical guidance for industrial production of cultured meat.

## 2. Materials and Methods

### 2.1. Cell Culture and Reagent

The cells used in all experiments were isolated from healthy pigs (piglets aged 3 to 7 days). All animal care and experimental protocols were approved and carried out in accordance with the Animal Care and Use Committee of Nanjing Agricultural University. In this part of the experiment to study the effects of long-term passage on SMCs, primary SMCs were cultured by continuous passage, and P2, P4, P6, P8, P10, P12 and P14 samples were collected for the determination of indexes. The cells frozen in liquid nitrogen were resuscitated and cultured in a collagen-covered culture dish at 37 °C under 5% CO_2_. The culture method of SMCs is as described previously [30]. The proportion of SMCs proliferation medium was DMEM/F12 (Gibco, Carlsbad, CA, USA) supplemented with 15% fetal bovine serum (Thermo, Waltham, MA, USA), 1% (*v*/*v*) penicillin/streptomycin (Gibco, USA) and the addition of a final concentration of 5 ng/mL basic fibroblast growth factor (Genscript, NKC, Zhenjiang, China). The SMCs’ differentiation medium was based on the proliferation medium with serum reduced to 2% and no basic fibroblast growth factor added. In order to study the effect of TGF-β1 on the function of late passage cells, passage 14 of cells was used for the experiment and adding 5ng/mL of TGF-β1 (GenScript, NKC, China) for a proliferation and differentiation medium for the culture of SMCs. The morphologic images of the cells were observed daily with a bright field microscope (Olympus, Tokyo, Japan), and the cell proliferation was recorded during the passage.

### 2.2. Quantitative Real-Time PCR

In this study, real-time quantitative PCR was used to detect the gene expression of related proteins in cells under different states. The sample harvesting method of real-time quantitative PCR is consistent with that described in previous research [31]. The total RNA of smooth muscle cells was collected and extracted according to the instructions. RNA concentration and purity were determined using Nano Drop2000 (Agilent Technologies, Santa Clara, CA, USA). RNA was reverse-transcribed into cDNA using a qScript cDNA Super Mix kit (TAKARA, QKV, Japan) according to the manufacturer’s instructions. The cDNA, primers of corresponding genes and reagents needed for PCR were configured into the machine system according to the manufacturer’s instructions. RT-qPCR was conducted using CFX Opus (BIO-RADXR, Hercules, CA, USA) and performed according to the amplification procedure specified in qPCR RT kit. The gene expression levels of the following proteins were determined: muscle proteins (actin, myosin heavy chain (MyHC)), proteins associated with SMCs differentiation (smoothelin) and extracellular matrix proteins (collagen III, collagen I, laminin, elastin and fibronectin). The relative differences in each sample were corrected using GAPDH mRNA as an internal control and normalized to the control level by the 2^−ΔΔCt^ method. Finally, the relative gene expression data were imported into GraphPad Prism for mapping.

### 2.3. Western Blot Analysis

The protein expression of SMCs was determined by Western blots. The sample collection and Western blots methods were performed according to Tom Ben-Arye [32]. The protein sample collection method for two-dimensional cells is as follows: suck up the culture medium of the cells in the Petri dish and add an appropriate amount of phosphate-buffered saline (PBS) for cleaning. Clean it three times to ensure that the residual culture medium is removed. Slightly dry PBS in a Petri dish and add precooled radioimmunoprecipitation assay (RIPA) lysis buffer (Beyotime, CGO, Shanghai, China) plus 1% protease inhibitors (Beyotime, CGO, China) to lyse cells for proteins collection in cells. The expression of muscle protein (actin, MyHC), phenotypic protein (smoothelin), extracellular matrix protein (collagen I, collagen III) and GAPDH (control) was detected in SMCs. An equal amount of total protein (20 μg) was loaded onto a 4–20% SDS-PAGE gel. After electrophoresis, the SDS-PAGE gels were placed on a wet transfer device for operation. And the proteins were then transferred to polyvinylidene fluoride (PVDF) membranes (GenScript, CGO, China). At the end of the transfer, the nitrocellulose membrane cut out the target band according to the knowledge of protein markers and put it into 5% skim milk powder for sealing at room temperature for two hours. After the closure of milk powder, the membranes were rinsed three times with TBST buffer (Tris, NaCl, Tween-20) and then incubated for 12 h at 4 °C with primary antibodies. The primary antibodies used in this experiment are as follows: mouse anti-GAPDH (1:1000; Millopore, Burlington, MA, USA), rabbit anti-α-SMA (1:500; Abcam, EGSC), mouse anti-smoothelin (1:200; Abcam, EGSC), mouse anti-MyHC (1:500; Invitrogen, Carlsbad, CA, USA) and mouse anti-collagen III (1:500; Santa Cruz Biotechnology, Dallas, TX, USA). After the incubation of the primary antibody, the primary antibody was recovered, and the membranes were cleaned three times with TBST. The second antibody was then added to the membrane and incubated at room temperature for 2 h. The horseradish peroxidase-conjugated goat anti-mouse IgG (1:2000) (CWBio, Taizhou, China) and horseradish peroxidase-conjugated donkey anti-rabbit IgG (1:2000) (CWBio, Taizhou, China) are the secondary antibodies used. After incubation, the membrane was colored with horseradish peroxidase chemiluminescence detection kit (Beyotime, CGO, China), and the expression of related proteins was detected with protein imaging system (Bio-Rad, CA, USA).

### 2.4. Immunocytochemistry

The expression of extracellular matrix proteins and muscle protein in SMCs was measured by immunofluorescence technique. At first, the two-dimensional cultured cells were fixed with 4% paraformaldehyde at 4 °C, and the samples were preserved at 4 °C. After the fixative is removed, the cells are cleaned three times with PBS, and the action should be gentle. And then cells were perfused with 0.5% Triton X-100 (PBS configuration) at room temperature for 15 min. After the transparency is completed, the cells were cleaned again and then incubated with primary antibody (actin, collagen III, DAPI) at 4 °C overnight. After the incubation of the primary antibody, the antibodies were recovered and washed with PBS five times. And then the cells were incubated with Goat anti-rabbit IgG secondary antibody (1:1000) (British Abcam, EGSC) and Goat anti-mouse IgG secondary antibody (1:1000) for 1 h at room temperature (Leica TCS sp8x, Weztlar, Germany).

### 2.5. Sirius Red Staining

The two-dimensional cultured cells were washed three times with PBS and fixed with 4% paraformaldehyde. The fixed sample was washed three times with PBS and then incubated with Sirius red staining solution for one hour away from light at room temperature. The incubated samples were washed three times with ultra-pure water and observed directly by microscopy. The image was taken with a microscope (Olympus, TKY, Japan).

### 2.6. Transcriptomics

The sample processing and collection methods of transcriptomics were performed according to Bonardi [33] with slight modification. When the cells were cultured to 80% confluence, the medium was drained, and the cells were washed three times with PBS. After PBS was dried, TRIzol reagent (Ambion, TX, USA) was added to collect RNA samples. Then, the total RNA of SMCs was collected and extracted according to the instructions. Nano Drop2000 (Agilent Technologies, CA, USA) was used to determine the integrity and amount of RNA prior to sending RNA sequencing. Passage 2 and passage 12 SMCs were used for cell RNA sample collection. 

### 2.7. Proteomics

Proteomic sample processing and collection was conducted as required by the sequencing company. After removing the medium, the cells in the culture dish were cleaned three times with PBS, and then the appropriate amount of PBS was added to hang the cells down with the cell scraper and collect them into the centrifuge tube. Collected cell samples were stored in a −80 °C refrigerator until sequencing.

### 2.8. Statistical Analysis

The statistical analysis used SAS 8.0 (SAS Institute Inc., Cary, NC, USA), and graph production used GraphPad Prism 5. The *t*-test and one-way ANOVA were used to calculate the difference between different treatments. The data were considered significantly different if *p* < 0.05.

## 3. Results and Discussion

### 3.1. Effect of Long-Term Subculture on SMCs Function In Vitro

**Figure 1:**
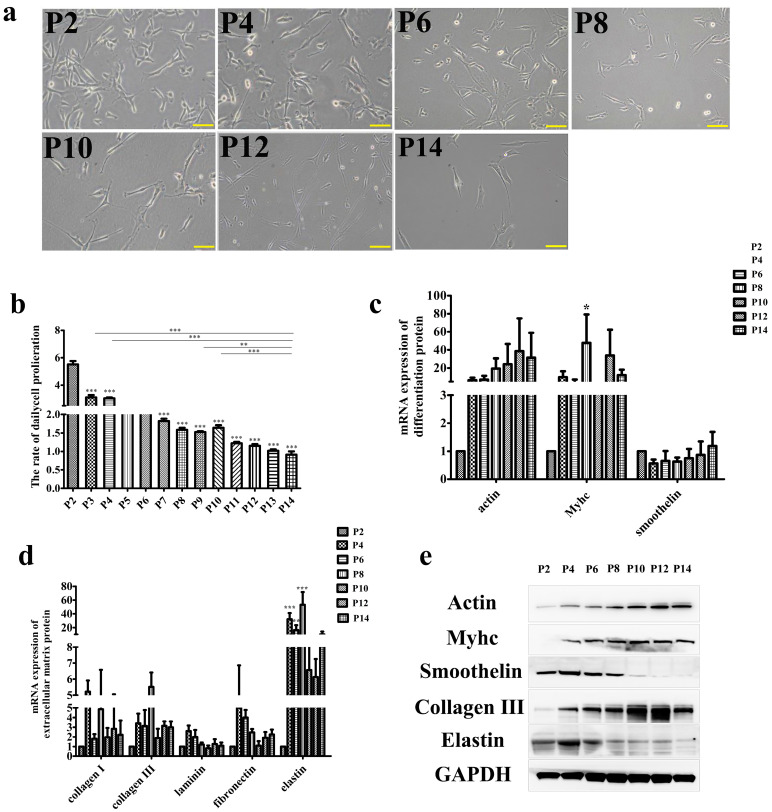
Effect of long-term subculture on functional characteristics of SMCs. (**a**) Bright field diagram of smooth muscle cells during passage. (**b**) Proliferation rate of smooth muscle cells in vitro. (**c**,**d**) Gene expression of phenotypic proteins and extracellular matrix proteins in smooth muscle cells during passage. (**e**) Protein expression levels of phenotypic proteins and extracellular matrix proteins in smooth muscle cells during passage. The length of scale in the bright field diagram is 100 μm. In the statistical results, * represents *p* < 0.05, ** represents *p* < 0.01, and *** represents *p* < 0.001.

Cultured meat companies need to expand their production scale to further commercialize. The expansion of cell culture is an important point to further promote the commercialization of cultured meat. This means that cells need to be cultured on a large scale in vitro. At present, the containers used to culture cells in vitro mainly include two-dimensional Petri dishes and three-dimensional microcarriers, but their surface areas are limited, so cells must be passaged to harvest more cells [34,35,36]. Therefore, we further studied the damage of long-term subculture on SMCs function and mastered the growth rhythm of SMCs during culture in vitro to provide theoretical support for the use of SMCs to produce cultured meat. The changes of SMCs morphology, multiplication factor, gene expression and protein expression after long-term culture are shown in Figure 1. The cell bright field diagram (Figure 1a) revealed that with the increase of cell passage times, the morphology of cells in the early stage of passage was mostly small in area and clear in edge outline, while in the late stage of passage, cells began to spread out in a larger area and the edge outline was not clear. From the point of view of cell morphology, we believe that cells with smaller area in the early stage of passage are in better condition, while cells in the late stage of passage are more damaged in vitro, so they show the state of aging cells with an unclear edge contour [37]. Then, the trypan blue counting method was used to measure the proliferation ability of SMCs in the process of cell passage (Figure 1b). It was found that the proliferation ability of SMCs decreased significantly (*p* < 0.05) with the increase of passage times, and the proliferation of SMCs basically did not occur at passage 14. This suggests that long term subculture in vitro may cause partial cell damage and decrease the proliferation ability of SMCs. The gene expression of phenotypic proteins of SMCs and the extracellular matrix protein in SMCs during the passage was further measured (Figure 1c,d). The results showed that the gene expression of MyHC was significantly up-regulated in SMCs at the later stage of passage, the gene expression of elastin was significantly up-regulated and then decreased, and the initial expression of passage 10 was no different from that of the previous generation (*p* < 0.05). The effect of passage on the expression of muscle protein and extracellular matrix protein in SMCs was determined. The results of Western blotting (Figure 1e) showed that with the increase of passage times, the expression of smoothelin decreased and the expression of actin and MyHC increased significantly, which means that continuous long-term passage caused SMCs to lose their contractile phenotype and change into a synthetic phenotype [38], accompanied by the secretion of large amounts of collagen. However, after cell passage to passage 14, synthetic SMCs produced a large amount of contractility protein, and collagen expression decreased again. This proved that SMCs became the synthetic phenotype at the initial stage of in vitro culture, and continued passage of SMCs with this phenotype would damage part of its function. The above results showed that the functional properties of the cells declined after long-term culture in vitro, and the same phenomenon was observed in other seed cells of cultured meat [39]. In addition, during the process of cell culture in vitro, the function of cells will be damaged due to experimental operations such as pancreatic enzyme action during multiple passages, fluid shear force during centrifugation, and the temperature effect of cell cryopreservation and recovery. Cultured meat products are mainly made from cells and cell products, and the total amount of cells and cell products determines the yield of cultured meat [40]. The decline in the function of seed cells during culture means that the production of animal protein decreases, thus reducing the yield of cultured meat products. Therefore, improving the protein production of cells during in vitro culture is also one of the focuses of research. 

### 3.2. Transcriptomic Analysis of the Effect of Long Passage on SMCs

**Figure 2:**
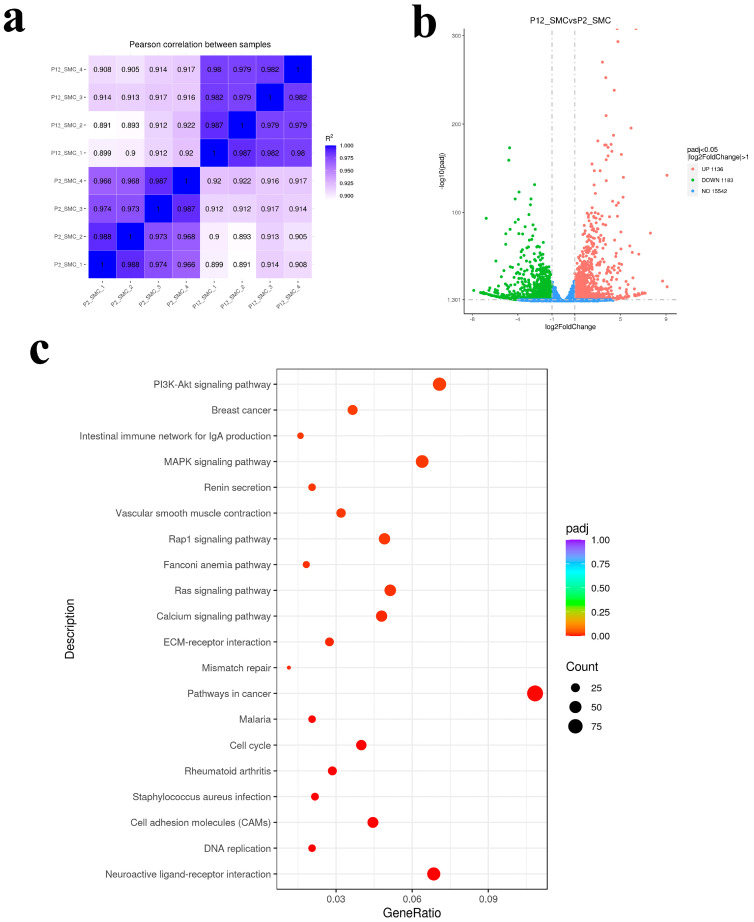
Transcriptomic analysis of smooth muscle cells in early and late passage. (**a**) Correlation heat maps of all samples. (**b**) Volcano plot of statistical significance (− log10 *q*-value) against enrichment (log2 fold change) of differentially expressed genes in passage 12 against passage 2. (**c**) The bubble plot of KEGG pathway analysis.

**Figure 3:**
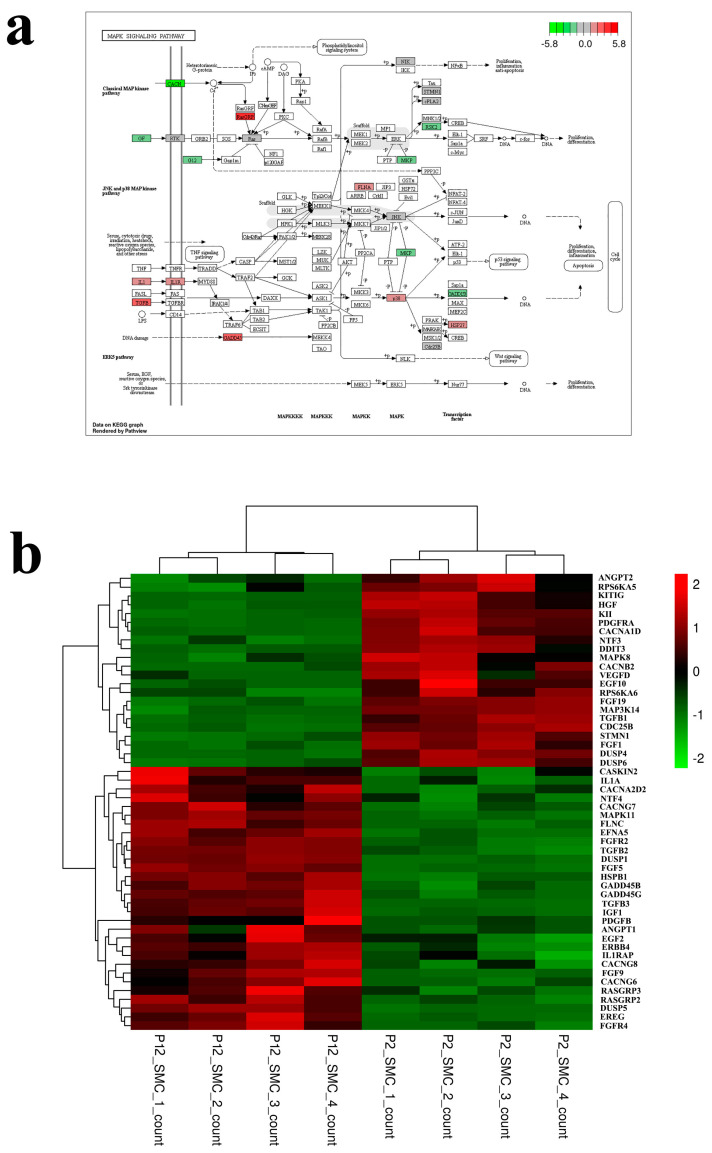
Changes of MAPK signaling pathway. (**a**) Map of MAPK signaling pathway after long-term passage of smooth muscle cells. (**b**) Heatmap of transcriptomic expression data showing differentially expressed genes in MAPK pathway.

**Figure 4:**
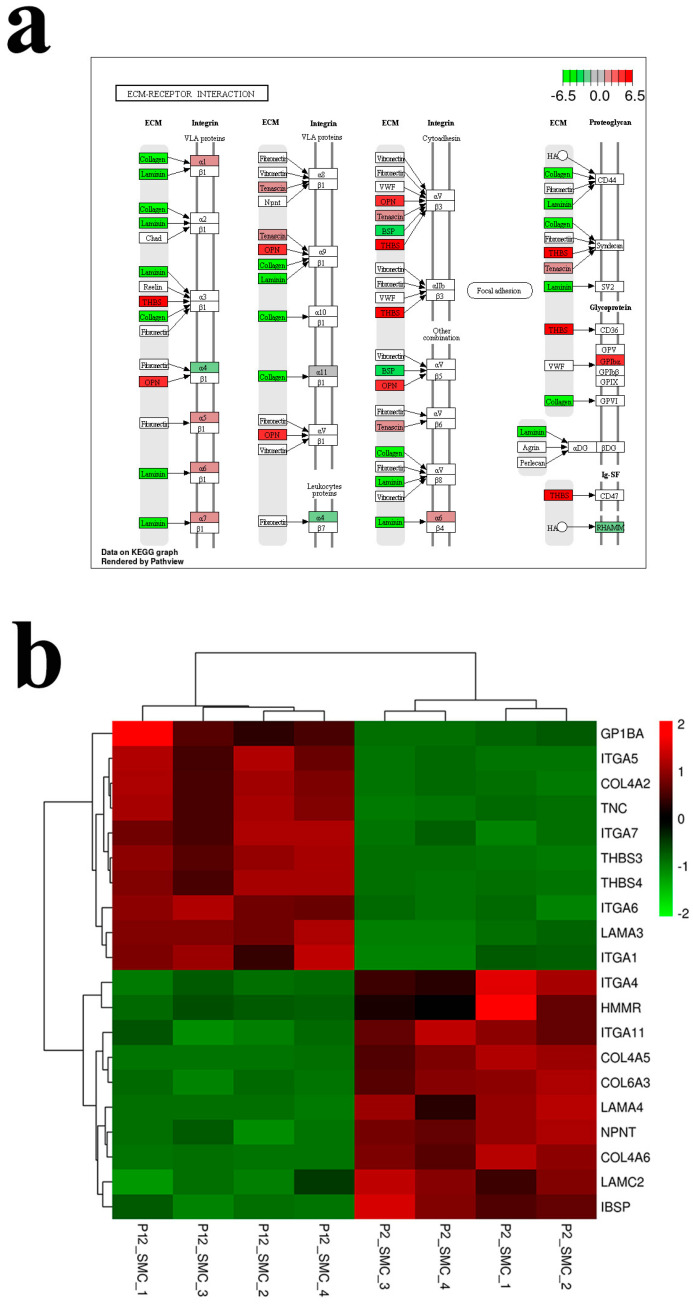
Changes of ECM-RECEPTOR INTERACTION signaling pathway. (**a**) Map of ECM-receptor interaction pathway after long-term passage of smooth muscle cells. (**b**) Heatmap of transcriptomic expression data showing differentially expressed genes in the ECM-receptor interaction pathway.

In order to explore the specific pathway through which long-term subculture damages SMCs in vitro, we conducted transcriptomic analysis of cells in the early and late stages of passage. The results in Figure 2 showed that the transcriptomic samples had good repeatability, with a total of 2319 differential genes, of which 1136 genes were up-regulated and 1183 genes were down-regulated. KEGG enrichment analysis of differential proteins revealed that differential genes were mainly concentrated in aging related signaling pathways, among which PI3K-Akt and MAPK signaling pathways were the most enriched genes [41]. The heat map and signal path map (Figure 3a,b) of MAPK pathway differential gene enrichment showed that the gene expressions of MAPK pathway receptor proteins RAS and TGF-β were up-regulated. Aging causes and is associated with abnormal function of multiple signaling pathways and many factors that maintain cell health [42]. The activation of mTOR increases the protein synthesis of MKK6 and enhances the activation of the p38 MAPK-p53 pathway leading to cell senescence [43,44]. Aging cells form oxidative stress due to the accumulation of reactive oxygen species, thus activating PI3K and mTOR and forming aging-related secretion phenotypes through the PI3K/mTOR signaling pathway [45,46]. This indicates that SMCs cultured in vitro with synthetic phenotype will also cause cell senescence after long-term passage. Meanwhile, some up-regulated genes were enriched in the ECM-receptor interaction pathway. Heat maps and pathway maps of ECM pathway (Figure 4a,b) enriched genes also showed down-regulated expression of the collagen receptor response factor gene in SMCs at the later stage of passage senescent cells when they begin to secrete some inflammatory proteins, showing a secretory senescence phenotype, and produce fewer extracellular matrix proteins [47]. The cells lose rigidity and become borderless. This is mainly due to increased activity of matrix metalloproteinases and impaired signaling of TGF-β induced by reactive oxygen species produced during aging [48,49,50]. Therefore, the results of transcriptomics suggest that long-term passage activates the senescence related pathways of SMCs and down-regulates the gene expression of extracellular matrix proteins. The possible reason is that oxidative stress of aging cells affects the signaling of the TGF-β pathway, which provides direction for improving the ability of SMCs to secret extracellular matrix proteins in the late passage.

### 3.3. TGF-β1 Promotes the Ability of SMCs to Secrete Collagen in Late Passage

**Figure 5:**
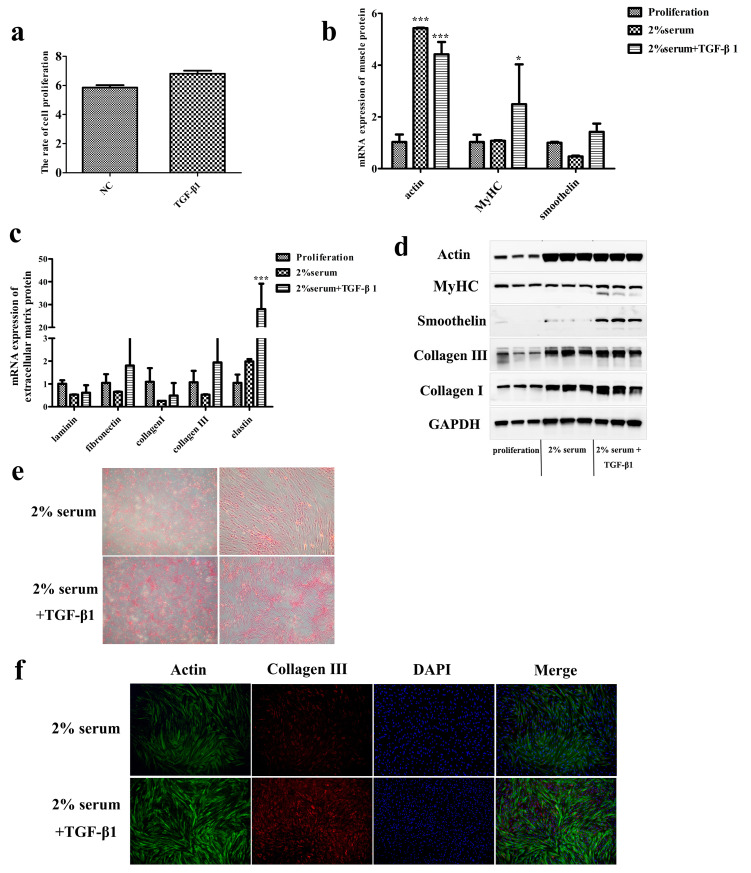
Effect of TGF-β1 on smooth muscle cells in late passage. (**a**) The proliferation rate of smooth muscle cells treated with TGF-β1 during the proliferative stage. (**b**,**c**) Gene expression of phenotypic proteins and extracellular matrix proteins in smooth muscle cells treated with TGF-β1 during the differentiation stage. (**d**) Protein expression levels of phenotypic proteins and extracellular matrix proteins in smooth muscle cells treated with TGF-β1 during the differentiation stage. (**e**) Sirius red staining of smooth muscle cells. (**f**) Immunofluorescence image of smooth muscle cells. In the statistical results, * represents *p* < 0.05, and *** represents *p* < 0.001.

The proliferation of SMCs slowed down at the later stage of passage; the production of collagen did not change after the induction of low serum; and the plasticity of cells declined. Research evidence confirms that TGF-β1 is a major regulator of ECM accumulation and therefore may be key to ameliorating the decline in extracellular matrix proteins secreted by aging SMCs in late passage [51]. In order to improve the secretion capacity of SMCs in late passage, TGF-β1 was added at the differentiation stage in order to make the cells in late passage secret more collagen to achieve the purpose of improving the texture of cultured meat in the production process. The results in Figure 5 showed that TGF-β1 treatment did not increase the proliferation rate of SMCs in the late passage stage. Real-time quantitative PCR and Western blotting were used to determine the effect of TGF-β1 on gene and protein expression of muscle proteins and extracellular matrix proteins in SMCs at the later stage of passage. The results of RT-qPCR (Figure 5b,c) showed that TGF-β1 significantly promoted the gene expression of actin, myosin and elastin in SMCs. The quantitative results of protein (Figure 5d) also showed that TGF-β1 promoted the protein expression of myosin, collagen type I and collagen type III. This means that the ability to secrete extracellular matrix proteins increased after SMCs were treated with TGF-β1 at the differentiation stage, as well as the expression of muscle proteins. Meanwhile, the addition of TGF-β1 significantly promoted the expression of smoothelin in the differentiation stage. Smoothelin was found only in fully differentiated contractile SMCs. They are increasingly used to monitor the process by which SMCs differentiate into systolic or synthetic phenotypes [52]. This means that SMCs in the late passage may change from synthetic phenotype to contractile phenotype in response to TGF-β1 and also express more phenotypic proteins that can be named muscle proteins. The results of Sirius red staining (Figure 5e) and immunofluorescence staining (Figure 5f) were found to be consistent with the results of protein Western blotting. The fluorescence intensity of collagen staining in the TGF-β1 treated group was stronger than that in the control group. In conclusion, TGF-β1 can not only promote the secretion of collagen in SMCs at the late stage of passage but also promote the expression of muscle proteins. The structure of traditional meat is made up of muscle fibers, adipose tissue and a network of connective tissue. Collagen is the main component of connective tissue in traditional meat, giving it its background toughness. This property of TGF-β1 that promotes SMCs to produce more collagen can be applied to produce cultured meat with customized texture using SMCs. In order to further mimic the natural structure of meat, muscle stem cells, fat cells and ECM protein-producing cells (such as fibroblasts, smooth muscle cells, etc.) may be co-cultured to produce products with structure and sensory flavor closer to traditional meat [14]. Moreover, TGF-β1 promotes the secretion of collagen in SMCs, which in the co-culture system containing SMCs and muscle stem cells can further promote the adhesion, migration and growth of other cells such as muscle stem cells. Therefore, for the cultured meat product itself, the study of TGF-β1 promoting production of collagen in SMCs can also be further applied to co-culture systems. 

### 3.4. Proteomic Analysis of the Effect of TGF-β1 on SMCs

**Figure 6:**
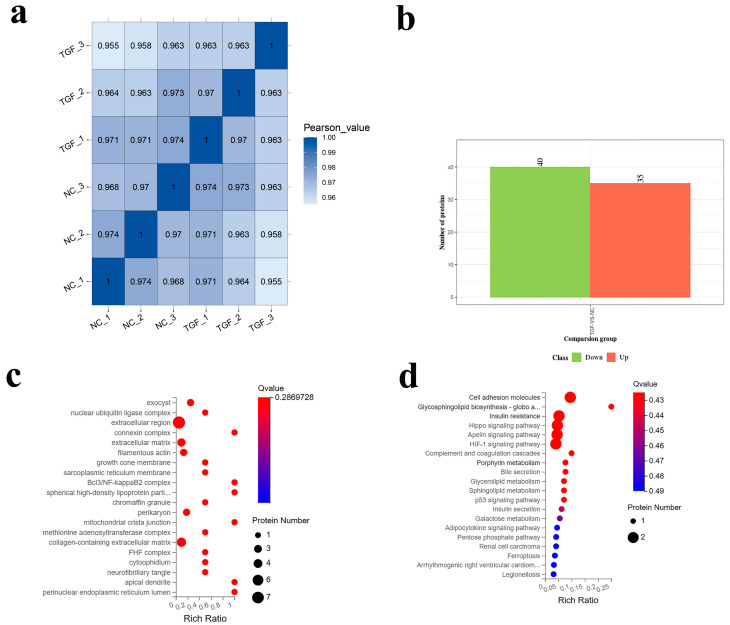
Proteomic analysis of smooth muscle cells treated with TGF-β1 at the late stage of passage. (**a**) Correlation heat maps of all samples. (**b**) Statistical results of differential proteins. (**c**) GO enrichment analysis of up-regulated proteins. (**d**) The bubble plot of KEGG pathway analysis.

Senescent cells begin to secrete a large number of inflammatory and proteolytic factors and appear a state of functional damage [53,54,55]. The research shows that TGF-β1 is also involved in regulating complex inflammation and generally considered to be an inhibitor of excessive inflammation [56,57,58]. Therefore, what way TGF-β1 promotes collagen secretion of SMCs at the later stage of passage was further analyzed by proteomics technology. The results of proteomics (Figure 6b) showed that there were 75 different proteins, of which 35 proteins were up-regulated and 40 proteins were down-regulated. Then, GO enrichment analysis (Figure 6c) was performed for differential proteins, and the results showed that differential proteins were mainly enriched in extracellular region, collagen-containing extracellular matrix proteins, extracellular matrix proteins and filamentous actin and other proteins. This result supports our previous conclusion that TGF-β1 can promote not only extracellular matrix protein secretion of SMCs in the late passage but also secretion of muscle protein. Then, KEGG pathway enrichment analysis (Figure 6d) was used to further analyze the differential proteins, and the results proved that these differential proteins were mainly enriched in cell adhesion molecules, insulin resistance, the Hippo signaling pathway, the Apelin signaling pathway and the HIF-1 signaling pathway. These results indicated that TGF-β1 greatly promoted the expression of extracellular matrix proteins mainly through the activation of cell adhesion molecular pathways. In addition, the Hippo pathway affects transcriptional programs important for cell proliferation, survival and migration through YAP/TAZ phosphorylation activation and YAP/TAZ dephosphorylation shutdown [59,60,61]. It has been reported that YAP/TAZ also increases miR-130/301 expression in pulmonary vascular cells to induce collagen deposition and Lox-dependent matrix remodeling [62]. Therefore, this indicated that TGF-β1 promoted Hippo pathway activation and inhibited cell proliferation and may further affect collagen formation through the Hippo pathway. Similarly, hypoxia-inducing factor-1α (HIF-1α) is thought to promote fibroblast differentiation and extracellular matrix protein deposition [63]. These results indicate that TGF-β1 treatment can promote the expression of cell adhesion molecules which activate the Hippo signaling pathway and HIF-1 signaling pathway and further promote the production of extracellular matrix proteins. 

## 4. Conclusions

This study aimed to explore the effect of long-term passage in vitro on porcine SMCs and the effect of TGF-β1 on porcine SMCs in the late passage, and further proteomic techniques were used to explore the potential mechanism of TGF-β1 action on SMCs. These works have proved that multiple passages would decrease the proliferation rate and differentiation ability of SMCs during the proliferation stage and differentiation stage. Long-term passage activates the senescence related pathways of SMCs and down-regulates the gene expression of extracellular matrix proteins. In addition, TGF-β1 can promote the secretion of collagen and muscle proteins in SMCs at the late stage of passage. The results of proteomics imply that TGF-β1 can promote the expression of cell adhesion molecules which activate the Hippo signaling pathway and the HIF-1 signaling pathway and further promote the production of extracellular matrix proteins. These results provide theoretical support for SMCs to be better used as seed cells of cultured meat, so as to better help the industrial production of cultured meat.

## Data Availability

The data presented in this study are available on request from the corresponding author. The data are not publicly available due to corresponding author requirement.
